# Wasting, Stunting, and Anemia in Angolan Children after Deworming with Albendazole or a Test-and-Treat Approach for Intestinal Parasites: Binary Longitudinal Models with Temporal Structure in a Four-Arm Randomized Trial

**DOI:** 10.3390/nu14112185

**Published:** 2022-05-24

**Authors:** Carolina Gasparinho, Maria Helena Gonçalves, Assucênio Chissaque, Giovani L. Silva, Filomeno Fortes, Luzia Gonçalves

**Affiliations:** 1 Centro de Investigação em Saúde de Angola (CISA), Rua Direita de Caxito, Caxito, Angola; 2 Global Health and Tropical Medicine (GHTM), Instituto de Higiene e Medicina Tropical (IHMT), Universidade Nova de Lisboa (UNL), 1349-008 Lisbon, Portugal; assucenio.chissaque@ins.gov.mz (A.C.); filomenofortes@ihmt.unl.pt (F.F.); 3 Departamento de Matemática, Faculdade de Ciências e Tecnologia, Universidade do Algarve, 8005-139 Faro, Portugal; mhgoncal@ualg.pt; 4 Centro de Estatística e Aplicações da Universidade de Lisboa (CEAUL), Faculdade de Ciências da Universidade de Lisboa, 1749-016 Lisbon, Portugal; giovani.silva@tecnico.ulisboa.pt; 5 Instituto Nacional de Saúde, Distrito de Marracuene, Maputo 264, Mozambique; 6 Departamento de Matemática, Instituto Superior Técnico, Universidade de Lisboa, Avenida Rovisco Pais, 1049-001 Lisbon, Portugal

**Keywords:** malnutrition, wasting, stunting, anemia, moderate-to-severe, mild-to-severe, longitudinal, deworming, test-and-treat, intestinal parasites, Angola

## Abstract

Undernutrition, anemia, and intestinal parasitic infections are public health problems in Angola, especially in pre-school children. We analyzed binary data from a longitudinal four-arm randomized parallel trial conducted in Bengo Province, northern Angola, over the course of two years, with seven follow-up assessments to explore the effects of four interventions (deworming and a test-and-treat approach for intestinal parasites, at both the individual and household levels) on wasting and stunting, and to understand their indirect benefits for anemia, malaria, diarrhea, and vomiting. A total of 121 children with intestinal parasitic infections received baseline treatment, and were allocated to the four arms (1:1:1:1). Using continuous outcome variables of height-for-age (HAZ) and weight-for-height (WHZ) statistical approaches did not reveal a clear benefit of any particular arm (Pathogens 2021, 10, 309). Next, HAZ and WHZ were transformed into binary variables of stunting and wasting, respectively, considering their mild-to-severe (Z-score < −1) and moderate-to-severe degrees (Z-score < −2). Original clinical data (on anemia, diarrhea, vomiting, and malaria) were also analyzed. From a binary longitudinal analysis with different dependence structures, using the R package *bild*, fitted models revealed the potential benefit of a test-and-treat approach at the individual level for wasting compared with annual albendazole at the individual level, especially considering mild-to-severe forms (OR_adj_ = 0.27; *p* = 0.007). All arms showed similar effects on stunting, compared with annual albendazole, at a 5% significance level. Time and age at baseline presented favorable effects in the percentage of stunting using both severity degrees. Results showed a decreased chance of having anemia and diarrhea over time, although with no significant differences between arms. Data from longitudinal studies are essential to study the direct and indirect effects of interventions, such as deworming, and to explore additional approaches aiming at better understanding the temporal structure of nutrition and health outcomes in children.

## 1. Introduction

Wasting (or acute malnutrition), stunting (or chronic malnutrition), and anemia are among the most important public health problems in children under five years of age, especially in low- and middle-income countries, where the health systems are most deprived [1,2]. Consequently, their reductions are implied in the 2025 Global Nutrition Targets, included in the second Sustainable Development Goal (SDG2) [3,4,5]. Despite some progress towards the global nutrition targets, it was estimated that 45.4 million (6.7%) children were moderately-to-severely wasted (weight-for-height (WHZ) < −2 Z-score) and 149.0 million (21.9%) were suffering from moderate-to-severe stunting (height-for-age (HAZ) < −2 Z-score) in 2020 [4]. In the African region, wasting was estimated to be slightly lower (6.0%) compared to the global levels, but the prevalence of stunting was considerably higher (30.7%) [4]. The World Health Organization (WHO) estimates that nearly 60.2% (95% confidence interval (95% CI) 56.6–63.7) of children under the age of five in the African region have anemia, which can result from nutritional deficiencies, hemoglobinopathies, and infectious diseases [6,7].

Several factors—such as diarrheal disease (representing 9.9% of child deaths) [8], civil war, extreme climate events, economic crisis, and poverty—can exacerbate the vulnerability of children to acute and chronic malnutrition, leading to a rapid change from mild (−2 ≤ Z-score < −1) to moderate (−3 ≤ Z-score < −2) or severe (Z-score < −3) forms. Studies focused on mild-to-severe forms are crucial to tackling the vulnerability of under-fives in the most deprived regions, where intestinal parasites also contribute to the vicious cycle of infection, anemia, malnutrition, and poverty [9]. Soil-transmitted helminths (STHs)—including *Ascaris lumbricoides*, *Trichuris trichiura*, and hookworms—are responsible for the infection of approximately 1.5 billion people worldwide through the contamination of the soil with eggs in human feces [9]. The vicious cycle of malnutrition and infection represents a huge public health problem, since it has a great impact on children’s development, causing an increased risk of infections, reduced school attendance, and compromised cognitive and physical development, with important impacts during their adulthood [9,10].

Angola is a sub-Saharan African country classified in the medium human development category [11]. However, based on different dimensions of poverty (i.e., health, education and living standards), 51.1% of the population is multidimensionally poor, distributed unequally in rural and urban areas (29.8% versus 88.2%, respectively) [12]. Insufficient data has been reported as one of the main problems in assessing the progress of the national nutritional indicators—especially the stunting target, which proposes a 40% reduction in the number of moderately-to-severely stunted children under five years of age by 2025 [5]. The most recent national nutritional survey refers to the period 2015–2016, and reported a very high prevalence of stunting (37.7%) and a lower prevalence of wasting (4.9%) in children under the age of five, including only their moderate-to-severe forms (Z-score < −2) [13]. Consequently, the magnitude of these percentages will be seriously amplified, including the mild forms of malnutrition. Moreover, a high prevalence of anemia in children under the age of five has also been reported at the national level (62.4%, 95% CI (50.0–72.7)) [14].

Deworming, or preventive chemotherapy with albendazole (ALB) or mebendazole, for children older than one year of age (along with other preventive strategies), has been recommended by the WHO to reduce the morbidity and the burden caused by STHs by reducing infection in endemic regions and increasing the weight and height gain of children [15,16]. In Angola, this strategy is included in the Neglected Tropical Diseases National Program, although it is mainly focused on school-age children, and with no available national data regarding pre-school children [17]. This public health strategy is applied irrespective of the presence of symptoms or infection (a test-and-treat (TT) approach is not considered) [16]; thus, it may neglect the existence and health impact of other parasitic infections, such as giardiasis, previously reported in Bengo Province [18]—an endemic region for STHs [19], with high levels of malnutrition [18,19] and anemia [18,19,20].

Thus, in this analysis we present an approach to explore the effects of different interventions on acute and chronic malnutrition outcomes (originally continuous outcomes converted into binary outcomes), in an attempt to take advantage of a two-year longitudinal four-arm randomized parallel trial previously conducted in a northern province of Angola (Bengo) [21]. More specifically, this longitudinal study with seven assessments aimed to investigate whether deworming with annual single-dose albendazole (annual-ALB) or a four-monthly test-and-treat (4TT) approach to intestinal parasites, at the individual or household levels, improved children’s nutritional outcomes [21]. In this paper, we aimed to explore the impact of allocated interventions on nutrition outcomes, by considering mild-to-severe and moderate-to-severe forms of malnutrition. Additionally, to understand the potential indirect benefits of this follow-up, data regarding anemia, malaria, diarrhea, and vomiting status were also analyzed.

## 2. Materials and Methods

### 2.1. Study Design, Setting, Sample Size, and Inclusion Criteria

**Figure A1:**
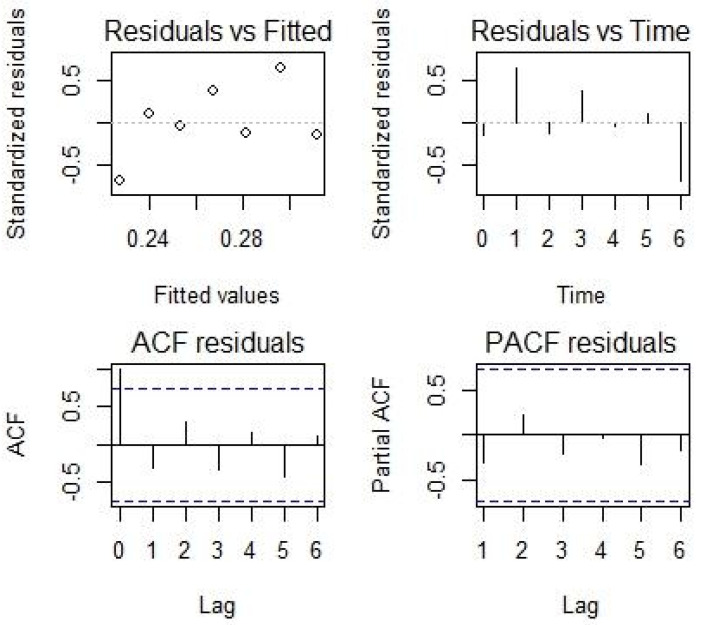
Residual analysis for model M8 considering mild-to-severe stunting.

This was a four-arm randomized parallel trial implemented in the Dande Health and Demographic Surveillance System (HDSS) study area of the Health Research Centre of Angola (CISA), located in the municipality of Dande, Bengo Province, Angola [21]. In a previous paper, Gasparinho et al. gave details regarding the sample size calculation (*n* = 151) based on HAZ (see [21] sub-section 2.9.1, Figure A1, and Appendix B). Participants were selected for inclusion between December 2013 and December 2014 according to the following criteria: (1) pre-school children infected with at least one pathogenic intestinal parasite, aged between 20 and 36 months at the time of recruitment, and less than 59 months of age at the end of the study; (2) residence in the HDSS area; and (3) no history of antibiotic or antiparasitic drugs in the previous 10 days.

Each arm of the study received a specific intervention:

- Arm 1 (A1): deworming with annual single-dose albendazole (400 mg) at the individual level (annual-ALB*individual level);
- Arm 2 (A2): deworming with annual single-dose albendazole (400 mg) at the household level (annual-ALB*household level);
- Arm 3 (A3): four-monthly test-and-treat approach to intestinal parasites at the individual level (4TT*individual);
- Arm 4 (A4): four-monthly test-and-treat approach to intestinal parasites at the household level (4TT*household level).

In two arms (A2 and A4), all household members were also invited to be included, whereas the remaining arms (A1 and A3) only considered the child in question. Before randomization and the intervention allocation, at baseline, children were treated with the standard treatment protocol in accordance with the parasitological diagnosis, as previously described [21]. In total, 121 participants were followed up in the community for two years, ending the study by January 2017.

### 2.2. Randomization Process, Baseline, and Follow-Up

**Figure 1:**
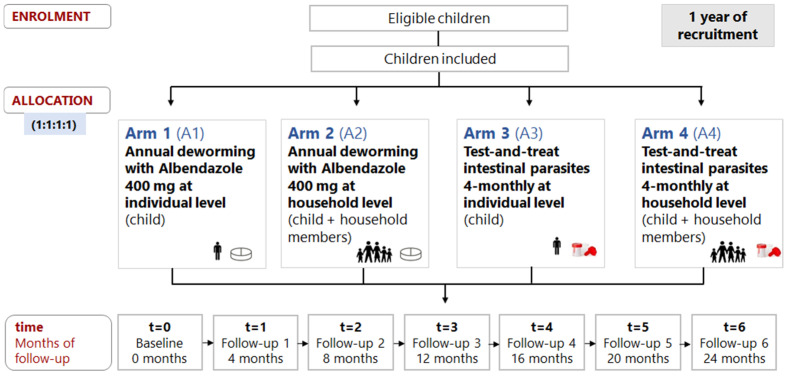
Randomization, allocation, and follow-up time of participants.

Children were randomly assigned to the four study arms at a ratio of 1:1:1:1 to receive the allocated intervention: A1 (n1 = 29), A2 (n2 = 31), A3 (n3 = 31), or A4 (n4 = 30) (Figure 1).

After baseline, a community follow-up (Fu) was performed at 4 (Fu1), 8 (Fu2), 12 (Fu3), 16 (Fu4), 20 (Fu5), and 24 months (Fu6). Children from arms A1 and A2 received annual-ALB (Fu1 and Fu4) at the individual and household levels, respectively, whereas a 4TT approach to intestinal parasites was performed in arms A3 and A4 at the individual and household levels, respectively.

More specifically, the 4TT approach included (1) the collection of one stool sample per child (A3) or per household member (A4) every four months for the diagnosis of pathogenic intestinal parasites, including protozoa and helminths (detailed laboratory methodology previously described in [21]), and (2) treatment of participants with positive results, according to the clinical protocol also described in Appendix A of the former manuscript [21].

Sociodemographic, water, sanitation, and hygiene conditions were collected at baseline. Anthropometric measurements were assessed by trained health professionals at baseline and at each follow-up time. Then, using WHO ANTHRO software (version 3.2.2, WHO, Geneva, Switzerland), HAZ and WHZ were standardized into anthropometric indices with Z-scores according to the groups of interest of this analysis, with different degrees of severity—(1) children with mild-to-severe stunting (HAZ < −1) and mild-to-severe wasting (WHZ < −1), and (2) children with moderate-to-severe stunting (HAZ < −2) and moderate-to-severe wasting (WHZ < −2) [22]. The following other clinical data were also obtained after a finger-prick blood sample collection: (1) hemoglobin concentration, to define anemia severity degree as no anemia (≥11.0 g per deciliter (g/dL)), mild (10.0–10.9 g/dL), moderate (7.0–9.9 g/dL), or severe anemia (<7.0 g/dL), using the HemoCue^®^ Hb 301 System (HemoCue^®^ AB, Angelholm, Sweden) [23]; and (2) malaria rapid immunochromatographic testing (Standard Diagnostics Bioline Malaria Ag P.f/P.v, Standard Diagnostics Inc., Giheung-gu, Korea). Reported clinical symptoms such as diarrhea and vomiting frequency were also obtained from caregivers.

### 2.3. Statistical Analysis

Following the Consolidated Standards of Reporting Trials (CONSORT) guidelines, intention-to-treat analysis included all randomized participants, after a missing data analysis of the continuous variables responsible for wasting and stunting in the present analysis [24]. For secondary outcomes, such as anemia, this was not performed, since it was not mandatory for the related data analysis. After an initial exploratory analysis, several binary longitudinal models were fitted to explain the potential effects of interventions, also considering the effects of time, age, and sex on stunting or wasting (our binary dependent variables) by using (1) the absence of a temporal dependence structure between follow-up times, meaning that the stunting or wasting status of a child at a particular moment does not depend on their stunting or wasting status in the previously observed moments of follow-up (this independence structure is an implausible situation); (2) Markov chain first-order dependence structure (MC1), meaning that the stunting or wasting status of a child at a given moment depends on their stunting or wasting status observed in the previous four months; and (3) Markov chain second-order dependence structure (MC2), meaning that the stunting and wasting status of a child at a given moment depends on the two previous moments of the follow-up (this means that their status is affected by their observed stunting or wasting status over the previous four and eight months).

Briefly, to illustrate how the models can be used in practice, we must present the case for the stunting outcome variable. Let $Y_{it}$ be a binary response, with $Y_{it}$ =1 if the $i^{th}$ child suffers from stunting (i = 1, …, 121) at the $t^{th}$ observation *t* = 0, …, 6. The marginal mean of the response (or the probability of a child suffering from stunting) is modelled as a logistic regression with the following covariates: (1) *arm* (a factor with 4 levels, where *arm1* (A1) is the reference class), (2) *time* = 0, 4, 8, 12, 16, 20, 24 months (*t* = 0 corresponding to baseline, *t* = 1 indicates 4 months of follow-up, …, *t* = 6 refers to 24 months of follow-up), and (3) age in months at baseline (*t* = 0). Mathematically, the marginal probability of a child having stunting ($P\left(Y_{it}=1\right)),$ which is related to the covariates through a logistic regression model, can be expressed as follows:

$$logit\;\left[P\left(Y_{it}=1\right)\right]=\beta_{1}+\beta_{2}\;arm2_{it}+\beta_{3}\;arm3_{it}+\beta_{4}\;arm4_{it}+\beta_{5}\;time_{it}+\;\beta_{6}\;age_{i}$$

where the regression parameters $\beta_{1},\ldots ,\;\beta_{6}$ in a marginal model (M) describe the effects of covariates on changes in the population’s mean response over time. For example, the estimated regression coefficients for *arm2* (A2), *arm3* (A3), and *arm4* (A4) (β_2_, β_3_, and β_4_, respectively) describe how the average proportion of stunting/wasting (hereafter also expressed in terms of odds ratios) would vary in the study population, comparing the intervention/treatment effects allocated in *arm2*, *arm3*, and *arm4* with *arm1*, while other covariates remain constant. Mathematically, the same modeling was used to explore whether anemia, malaria, diarrhea, and vomiting status were affected by interventions and/or by follow-up time. For this type of data, the presence/absence of a dependence structure may be expected/unexpected (e.g., anemia/malaria).

Models with different dependence structures were implemented using the *bild* package in R free software (R Core Team, Vienna, Austria) [25]. In order to illustrate the use of the *bild* package, an example for a particular model is presented in Appendix A.

Considering the dependent variables (stunting, wasting, anemia, malaria, diarrhea, and vomiting) for a type of dependence structure, and for a set of explanatory variables, there are several possible models to describe our data. The best models were selected based on the (1) Akaike information criterion (AIC), (2) Bayesian information criterion (BIC), and (3) log of the likelihood values (LogLike). These measures are widely used for model selection in health and biological modeling. However, different criteria sometimes support different models, leading to discussions about which is the most trustworthy. Additionally, residual analysis was performed for the selected model (Appendix B).

An alternative perspective on these criteria that can help in interpreting their practical implications is presented elsewhere [26]. The selected models are described in more detail, by presenting estimates of the fixed-effects parameters with standard errors (SEs), and converted into adjusted odds ratio (OR_adj_) values with *p*-values and 95% CIs to facilitate their interpretation.

## 3. Results

### 3.1. Baseline Characterization of Participants

**Figure 2:**
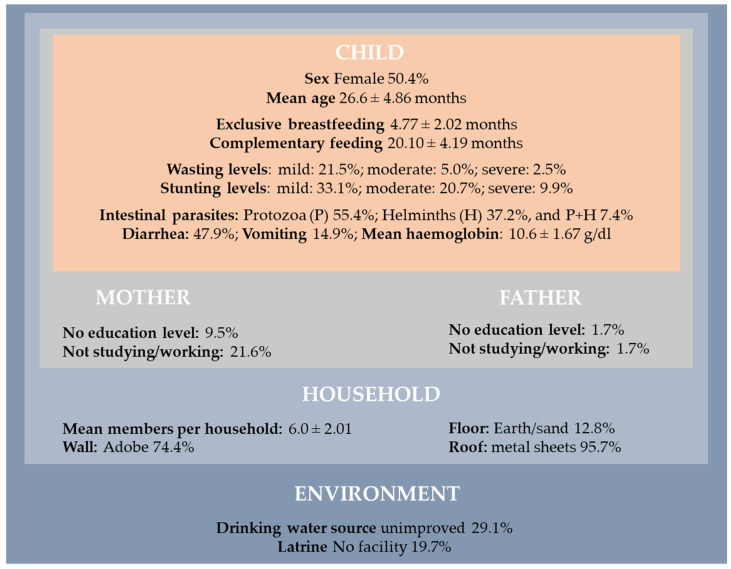
Baseline sociodemographic characteristics, nutritional status, and health conditions of the participants (*n* = 121, *t* = 0).

A detailed analysis of the baseline characteristics is provided in our original study [21]. Here, an overall description is presented to highlight the health and living conditions of the 121 children included at baseline (Figure 2).

In general, children were living in houses with several members per household (mean: 6.0; standard deviation = 2.01). Our findings revealed that 19.7% of the children were living in houses without latrines, and 29.1% with unimproved drinking water. Regarding the characteristics of their parents, 9.5% of the mothers and 1.7% of the fathers did not attend any level of education, and were not studying or working (21.6%: mothers vs. 1.7%: fathers). Mothers were in a disadvantaged situation.

In this poor environment, 55.4% of the children were infected by protozoan intestinal parasites and 37.2% by helminths, while 7.4% were infected by both at the baseline (Figure 2). Globally, children had single or multiple infections caused by *Giardia lamblia* (57.0%), *Ascaris lumbricoides* (25.6%), *Strongyloides stercoralis* (13.2%), *Trichuris trichiura* (5.8%), *Hymenolepis nana* (5.8%), *Cryptosporidium* spp. (5.8%), and *Entamoeba histolytica* (2.5%). Caregivers reported diarrhea and vomiting in 47.9% and 14.9% of included children, respectively. Anemia was diagnosed in 53.8% of infected children (30.3% with mild, 18.5% with moderate, and 5.0% with severe anemia). Considering nutritional status, 28.9% of children were suffering from mild-to-severe wasting (of whom 7.4% had moderate-to-severe levels) and 63.6% were stunted (of whom 30.6% were moderately-to-severely stunted).

**Figure 3:**
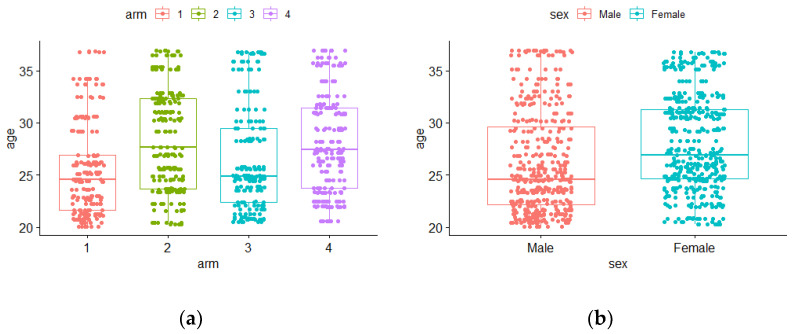
Boxplots for age at baseline by (**a**) arms: 1 to 4 and (**b**) sex: male or female.

Children’s ages (in months) by arm and sex at baseline are presented in Figure 3a,b, respectively.

In arms A1 and A3, the median age values seemed to be lower when compared with A2 and A4 (Figure 3a). An empirical difference in median age also occurred between males and females, with females having a higher median age (Figure 3b). Nevertheless, the hypothesis tests previously used did not show significant differences [21]. In this work, by applying binary longitudinal models, the effects of these variables (age, sex, arm, and all of them introduced simultaneously) on stunting and/or wasting status could be revealed (or not).

### 3.2. Mild-to-Severe and Moderate-to-Severe Stunting and Wasting

The numbers (*n*) and the percentages of children (%) with mild-to-severe and moderate-to-severe stunting and wasting are presented in Table 1 and Table 2, respectively.

As shown in Table 1, the percentage of children with mild-to-severe stunting was commonly more than twice the percentage of those with the moderate-to-severe forms, emphasizing the magnitude of the mild forms. More than 2/3 of the children at Fu1 (with a worse situation in A1—72.4%) presented mild-to-severe stunting, but by the end of the study (Fu6), that percentage decreased to below 50%—except in A3, where it was 54.8%. When removing the mild forms, the moderate-to-severe forms affected ¼ of the children in arms A1 and A3, and more than 1/3 in the arms including household members (A2 and A4). Overall, the percentage of moderate-to-severe stunting reduced from 30.6% at Fu1 to 22.3% at the end of the study (varying from 19.4% in A2 to 26.7% in A4).

According to Table 2, the existence of children suffering from wasting was essentially due to its mild form, since there were follow-up assessments where none of the children were suffering from moderate-to-severe wasting. Here, non-pattern values occurred over time, from a minimum of 17 (14.0%) to a maximum of 27 (22.3%) children detected with mild-to-severe wasting during the six follow-ups. Arm 1, at all follow-ups, presented the highest numbers of children with mild-to-severe wasting.

Regarding to moderate-to-severe forms, 1 (0.8%) to 5 (4.1%) children were identified in consecutive follow-ups (Fu2 and Fu3) (Table 2).

**Figure 4:**
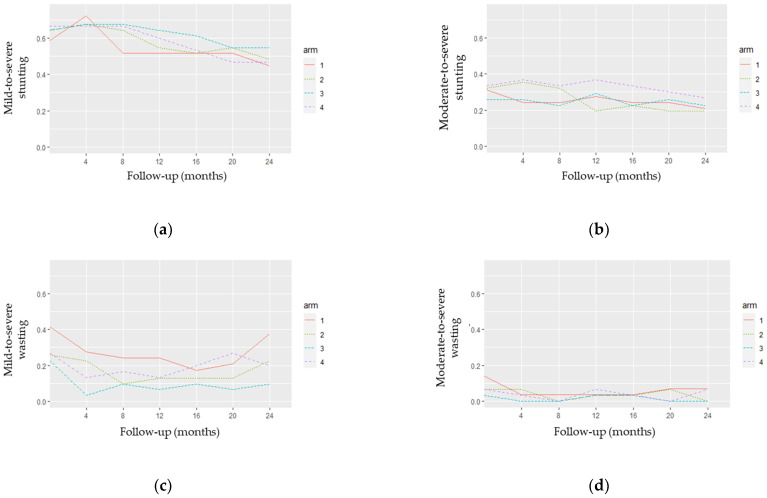
Observed stunting and wasting profiles by arm: (**a**) mild-to-severe stunting; (**b**) moderate-to-severe stunting; (**c**) mild-to-severe wasting; (**d**) moderate-to-severe wasting.

Figure 4 displays four plots related to observed stunting (Figure 4a,b) and wasting (Figure 4c,d) profiles, by arm.

Figure 4a,b show some crossover of the stunting profiles, which may indicate the non-distinction between their curves in both Z-score definitions. Figure 4c,d indicate some distinction between the wasting profiles, clearly differentiating arms A1 and A3 in Figure 4c. Furthermore, the plots in Figure 4a,b) suggest a decrease in the stunting curves over time. This pattern is not evident for the wasting curves (Figure 4c,d).

**Figure 5:**
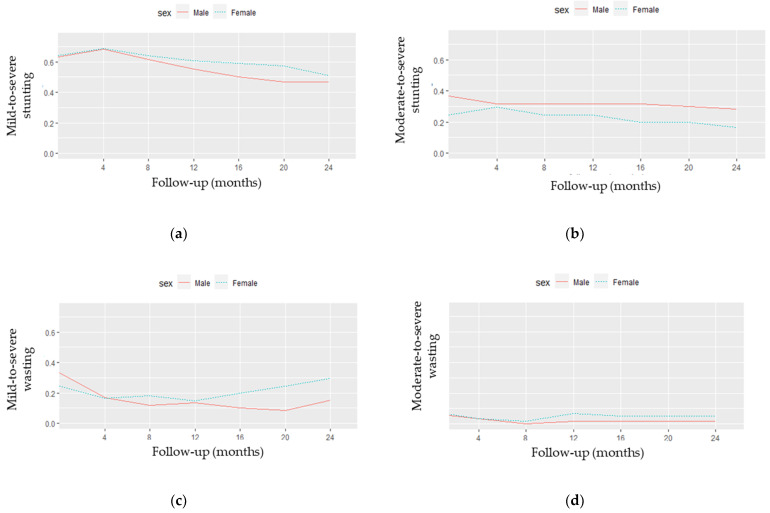
Observed stunting and wasting profiles by sex: (**a**) mild-to-severe stunting; (**b**) moderate-to-severe stunting; (**c**) mild-to-severe wasting; (**d**) moderate-to-severe wasting.

Figure 5 presents the observed stunting (Figure 5a,b) and wasting (Figure 5c,d) profiles by sex. As shown in Figure 5a,b, there seemed to be a distinction in the stunting curves between males and females after the fourth month of follow-up (Fu1). More specifically, the proportion of mild-to-severe stunting became higher in female than in male children after Fu1, while moderate-to-severe stunting levels were still more frequent in male children. Mild-to-severe wasting (Figure 5c) began higher in male children until the fourth month, when an inversion of the curves occurred. After 12 months of follow-up (Fu3), the proportion of mild-to-severe wasting seemed to increase in female children. Regarding Figure 5d, a smaller distinction between males and females with moderate-to-severe wasting was observed.

Table 3 provides the results of several models used to analyze stunting and wasting, according to arm, time, age, and sex, in the mentioned conditions (1), (2), and (3).

For stunting, models M7, M8, and M9 revealed the importance of second-order dependence (i.e., the last 8 months) for mild-to-severe and moderate-to-severe degrees of stunting. The AIC and LogLike values of the models M8 and M9 were very similar. The BIC measure tends to penalize models with a higher number of parameters. Thus, following the parsimony principle, the BIC values suggest M7 as the simplest model with a statistical fitting. Balancing biomedical information with statistical criteria, we opted to explore the model M8—which includes arm, time, and age for both stunting degree groups considered—in more detail.

In terms of wasting, among the nine models, again, the three models M7, M8, and M9 presented better fitting. However, in this case, the AIC and BIC values supported model M7, which was chosen to be explored in more detail. The LogLike values suggested M9 as the best model, but with similar values to M7 and M8. Detailed information on the selected models M8 (for stunting) and M7 (for wasting) is provided in Table 4.

Considering a second-order structure, the selected model for stunting (M8) for both groups of degree indicated that there were no significant differences between A1 (the reference group) and arms A2, A3, and A4. The estimated effects of time and age were significant at the 5% level for both groups of stunting (Table 4).

**Figure 6:**
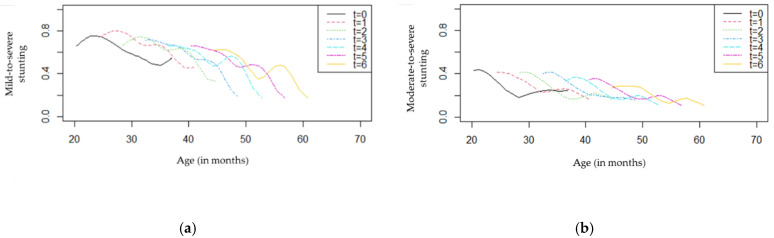
Stunting profiles by age and follow-up (*t* = 0,1,2,3,4,5,6): (**a**) mild-to-severe stunting; (**b**) moderate-to-severe stunting.

Since there was no arm effect on stunting, a graphical presentation of the overall numbers of children with different ages at baseline can be useful to illustrate the progress during the follow-up period (Figure 6).

As shown in Figure 6a, children with different ages seemed to present a decrease in the proportion of mild-to-severe stunting over time. At baseline, younger children had higher proportions of stunting (>50%) compared with older children, and this difference tended to be higher from eight months of follow-up (*t* = 2) to the end of the study (*t* = 6). Regarding Figure 6b, an overall reduction in moderate-to-severe stunting was observed for all ages. These proportions were lower than those presented in Figure 6a, as expected.

Returning to the selected models (Table 4), to quantify the changes in the proportion of mild-to-severe stunting, while keeping the arm and time constant, the estimated effect of time (OR_adj_ = 0.97, 95% CI (0.95, 0.99)) showed that for every four months of follow-up the odds of mild-to-severe stunting decreased by 3.0% ((1–0.97) × 100%); regarding age, a one month increase in age (OR_adj_ = 0.91; 95% CI (0.85, 0.97)) was associated with a 9.0% decrease in the odds of developing mild-to-severe stunting. Considering the selected model for moderate-to-severe stunting, a decrease of 2.0% in the odds of moderate-to-severe stunting for every four months of the follow-up was expected, along with a decrease of 9.0% in the odds of moderate-to-severe stunting for every one-month increase in the age of the child.

Table 4 also shows the estimated parameters of the selected model (M7) for wasting, with a significant difference between arms A3 and A1 for mild-to-severe forms (*p* = 0.007), whereas it was non-significant at the 5% level (*p* = 0.080) for moderate-to-severe forms. In the first case, the odds of a child suffering from mild-to-severe wasting in A3 were 73.0% lower when compared to children in A1 (OR_adj_ = 0.27; 95% CI (0.10, 0.69)), considering a fixed time. In terms of time, for both definitions, at the 5% significance level, M7 did not show a significant trend for wasting throughout the follow-up period.

These types of models also express additional findings of practical interest, since it is possible to calculate the estimated probability of a child suffering from wasting at a specific point in the follow-up. For example, according to M7, after eight months of the allocated treatment (time = 12 months), the estimated probability of a child suffering from mild-to-severe wasting was 0.313 (A1), 0.189 (A2), 0.109 (A3), and 0.207 (A4). At the end of the follow-up (time = 24 months), we obtained 0.276 (A1), 0.163 (A2), 0.093 (A3), and 0.179 (A4), highlighting better results for children in A3.

Regarding the importance of serial dependence, the two parameters (logpsi1 and logpsi2) associated with models M8 and M7 (Table 4) were significant at the 5% level, and were clearly non-zero, indicating a strong dependence based on MC2 for all selected marginal models. This means that stunting and wasting at a particular moment of the follow-up were correlated with the two previous assessments (around 8 months earlier).

### 3.3. Clinical Data: Anemia, Malaria, Diarrhea, and Vomiting

Table 5 and Table 6 show clinical data collected from participants at each follow-up.

According to Table 5, the percentage of children with anemia (Hb > 11 g/dL) at the baseline was the highest observed compared to any other follow-up assessment, regardless of the arm to which they were allocated (ranging from 41.9% in A3 to 63.3% in A4). After performing the allocated interventions, there were no effect differences on the anemia percentages in arms A2, A3, and A4 compared to A1. However, the chance of having anemia decreased over the follow-up period (from baseline to Fu6), with an overall reduction of 24.8%. By arm, this reduction in anemia was 26% in A1, 22% in A2, 15.0% in A3, and 37.4% in A4. We observed a second-order dependence for anemia, meaning that having anemia at a particular moment was correlated with at least the two previous assessments (8 months earlier).

Regarding malaria, all positive tests obtained were related to *P. falciparum*. No significant effects of the interventions or time on malaria frequency were observed. Simple models, without a dependence structure, seem to describe our data on malaria well.Regarding reported symptoms in children, Table 6 depicts a global reduction of 38.6% in diarrhea (from 47.9% at baseline to 9.3% at Fu6), with no different effects after performing the allocated interventions in arms A2, A3, and A4 compared to A1. The chances of having diarrhea also decreased over time by arm: 48% in A1, 24.9% in A2, 40.1% in A3, and 41.9% in A4. A first-order dependence structure for diarrhea was observed; in other words, reported diarrhea at a given time was correlated with the previous follow-up (4 months). Additionally, included children presented symptoms of vomiting at the baseline (14.9%), but no significant effect on vomiting was observed between arms, nor was there a longitudinal dependence structure with previous follow-ups.

## 4. Discussion

The first aim of this study was to investigate the impact of interventions (annual-ALB and a 4TT approach to intestinal parasites, at both the individual and household levels) on stunting (based on HAZ) and wasting (based on WHZ) of children infected with intestinal parasites and aged between 20 and 36 months at baseline in a longitudinal study with two years of follow-up. For the analysis of both nutritional outcomes, children were grouped considering mild-to-severe and moderate-to-severe levels, the latter commonly used by the WHO and in official indicators to motorize SDG2 [3,4,5]. Additionally, original clinical data assessed at baseline and during follow-up were also provided for anemia, malaria, diarrhea, and vomiting, since these are important health issues in African children.

### 4.1. Wasting and Stunting

This binary longitudinal analysis showed the potential benefit in reducing mild-to-severe wasting (OR_adj_ = 0.27; *p* = 0.007) after a 4TT approach to intestinal parasites at the individual level (A3), compared to annual-ALB (A1). However, this was not detected at the 5% significance level (*p* = 0.080) when considering moderate-to-severe wasting (a severity classification more commonly found in reports and scientific papers)—probably due to the reduced numbers of children at some follow-up times, among other reasons.

The higher levels of wasting at baseline could have been a result of a weight loss that had developed over a short period due to a recent episode of infection (in this case, the pathogenic parasitic infection detected in all participants at baseline and treated before allocated interventions) [27]. Non-pattern values of wasting occurred over time, with minimal fluctuations during the follow-up period, ranging between 14.0% and 22.3% for children with mild-to-severe wasting (and 0.8% and 4.1% when considering moderate-to-severe wasting). Similar to our results, no linear trend of wasting was detected over time in a secondary analysis of a longitudinal study conducted between February 2016 and August 2018, aiming to explore the temporal changes in wasting (and stunting) of Cambodian children recruited aged less than 24 months, and followed-up every 3–4 months for two years [28]. However, the mentioned study only considered moderate-to-severe wasting in its statistical analysis [28] and, therefore, our data regarding mild-to-severe wasting provide new findings for this unstudied vulnerable group.

Regarding the potential benefit of A3 compared to A1 for wasting, several factors could have contributed to this situation. For example, protozoan infections caused by *Giardia lamblia* were the most frequent in the included participants [21], highlighting the importance of thinking beyond deworming [29], which is only targeted at specific helminths and distributed to children without knowing their infection status, as recommended by the WHO [16]. Moreover, a high percentage of *G. lamblia* (37.9%, 95% CI (32.8–43.0%) has been also reported in school children in Cubal, western Angola, showing that this type of infection remains frequent in older children [30].

Given the logistic simplicity and low cost of delivering preventive chemotherapy (annual-ALB), it is routinely used in children to prevent and reduce STH infections in endemic regions [16]. However, divergent results of its benefits for child nutrition have been reported in the literature [31], which was one of the main reasons that motivated our research team to design this four-arm longitudinal trial in Angola [21], and to apply this additional statistical analysis. Current national guidelines for STH control are focused on school-based deworming [17]. Expanding deworming to all age groups has been noted as a benefit to communities in general and, in particular, to school-age children, by reducing their risk of reinfection [32]. Our study included a particular age group not included in the eligible priority age group established by national guidelines to receive the low-cost single dose [17]. In African settings such as Bengo, with the high risk of multiple parasites playing an important role in the progression and the severity of parasite-related disease (and, thus, increasing malnutrition levels) [9,33], a TT approach to intestinal parasites (not only for STHs, but also for protozoa) should be considered [34].

Despite being more expensive and demanding challenging field organization and laboratory staff training for ensuring data of high quality [35], this type of approach allows the appropriate treatment to be given to the child according to their previous diagnosis; thus, it is expected to be more effective in the long-term, and to improve the general condition of the child.

The global frequency rate of moderate-to-severe stunting was very high at baseline (30.6%) and, when adding children with mild stunting, it doubled (63.6%). This confirms that before the age of 20–36 months (the age inclusion criteria used in this study), children were already suffering the consequences of several factors extensively reported to impact their life at birth and in this early stage of life [36], thus recognizing stunting as an important public health problem that must be prioritized in Bengo.

Although most studies have failed to describe it, in our study we believe that in addition to reporting sex-specific analysis, our results provide new information by also adding mild levels of malnutrition. We observed a higher frequency of moderate-to-severe stunting in male children than in female children throughout the follow-up period. A male disadvantage in linear growth compared to girls has been previously reported, despite regional, national, and temporal variations also being described [37,38]. Our results are in accordance with a study reporting data from 87 cross-sectional Demographic and Health Surveys and Multiple Cluster Indicator Surveys 2010–2019 (including Angola) showing a significant male disadvantage [38]. Immunological factors leading to higher incidence of infectious diseases, along with cultural and economic factors—especially in poor countries—have been pointed out as some of the causes of the greater vulnerability of male children [38]. Similarly, a longitudinal study conducted in Cambodian children also reported a higher prevalence of stunting in males compared to females [28]. Additionally, in our study, when adding the mild form of stunting, we observed a female disadvantage compared to boys after the first follow-up (with children ranging between 24 and 40 months of age). Overall, it seems that male children are more vulnerable to more severe degrees of stunting, while mild forms are more frequent in female children.

In our study, age at baseline and time presented significant effects on stunting across the two years of follow-up. Conversely, no significant differences were obtained between arms. In general, it was observed that younger children had higher proportions of stunting at baseline compared with older ones, and the proportions progressively improved in all children across the two years of follow-up, irrespective of which arm they were allocated to—especially from Fu2 (eight months of follow-up) to the end of the study.

Changes in the proportions of mild-to-severe and moderate-to-severe stunting were quantified every four months of follow-up, with a decrease of 3% and 2%, respectively, while the same reduction was quantified for both groups of stunting severity for each one-month increase in the age of the child (9%). The selected models (with second-order dependence) reinforced that stunting and wasting at a given time may depend on the previous 8 months.

A global reduction in the percentage of children with mild-to-severe stunting was observed (decreasing from 68.6% at Fu1 to 48.8% at Fu6, but remaining more than twice the percentage of children with moderate-to-severe stunting at all follow-up times—from 30.6% to 22.3%, respectively).

Previous studies have suggested that wasting can contribute to an increased risk of subsequent stunting and, considering the same rationale, the aforementioned study conducted in Cambodia showed that interventions focused on wasting prevention and treatment can contribute to stunting reduction [28,36]. More specifically, the authors reported a catch-up linear growth induced by a correction of moderate-to-severe wasting in the previous three months [28]. This enhances the importance of longitudinal studies; more specifically, it makes clear how essential it is to follow-up children receiving nutrition-related interventions aiming to reduce malnutrition, and to describe and explore how nutritional outcomes vary across time and when considering other variables of interest.

### 4.2. The Importance of Not Neglecting Mild Forms of Malnutrition

Data reporting the mild levels of malnutrition are scarce in research, which tends to focus on moderate-to-severe levels. However, children with mild malnutrition assume a particular importance, as they can easily contribute to the burden of moderate and severe forms. In 2012, a paper published in *The Lancet* highlighted the need to assess all levels of mild-to-severe undernutrition [39], but little information on these trends is available [39]. From a nutritional perspective, this analysis allowed us to clearly identify the burden and the importance of mild levels of wasting and stunting in children in this study. For example, the magnitude and the fluctuation of mild-to-severe wasting (14.0–22.3%) were essentially due to its mild form. Moreover, this fluctuation expresses how weight can change between different moments of a child’s life, and makes it difficult to assess the progress of wasting over time. The contribution of mild levels of stunting highlights the importance of its inclusion when reporting nutritional outcomes—not only through different studies, but also when considering the progress of SDG2 across countries (which is only targeted at moderate-to-severe levels).

### 4.3. Anemia and Other Clinical Conditions

Regarding clinical data, it was possible to verify a decreased chance of having anemia and diarrhea over time, although with no significant differences between arms. The highest values for anemia were observed at baseline (53.8%), regardless of the arm to which the children were allocated. During the follow-up, overall anemia ranged between 36% (Fu1) and 22.1% (Fu4), ending the study with a total percentage of 29% (Fu6). The anemia values found at baseline were closer to those reported at the national level (62.4%) [14]. A similar prevalence of anemia (64.1%, 95% CI (63.9%, 64.4)) was reported in a secondary data analysis carried out based on the most recent Demographic Health Survey datasets collected in 32 sub-Saharan African countries from 2005 to 2018 [7]. Our models highlighted the importance of looking for long-term dependencies (at least eight months and four months) for anemia and diarrhea, probably reflecting the favorable environment of this geographic area. In addition to anemia and diarrhea, descriptive data also showed an overall decrease in malaria and vomiting. We consider that this reduction in the percentage of clinical conditions was expected. Of course, the interventions were not directly aimed at these health problems, but in some way their effects were indirectly anticipated, because all followed-up children received an allocated treatment expected to be more favorable compared to not receiving a treatment at all. Parasitic infections can lead to anemia. In our study, *G. lamblia* was the most common protozoan at baseline (previously reported to contribute to malabsorption of micronutrients), followed by *A. lumbricoides* (25.6%), which can cause iron-deficiency anemia [40].

### 4.4. Multiple Causes of Malnutrition and Indirect Health Benefits for Participants

The causes of malnutrition are multifaceted, and most of them are linked with social, economic, and political factors [2,41,42]. Several other factors beyond parasitic intestinal infections—such as the suboptimal feeding practices, the household food insecurity caused by climatic conditions and drought, the mothers’ levels of education, the inadequate access to clean water, sanitation, and hygiene (WASH), and the environment—could have contributed to the burden and the progress of malnutrition in our participants [27,41,42,43,44,45,46].

Regardless of our study’s results, given the diversity of factors leading to malnutrition, we believe that there may have been indirect health benefits for all participants. For example, in our study it was possible to verify a high percentage of mothers with no level of education (9.5%), which has been reported to impact on childhood wasting in other African countries, since the absence of knowledge on child health promotion can negatively influence the nutritional status of children ([43,47]. From this point of view, nutritional education targeting mothers with no formal education can be interesting for future interventions in child nutrition. For example, in poor communities in Bangladesh, a collaborative monitoring and evaluation system for a community-based nutrition project included multifaceted training for mothers of children aged between 0 and 59 months (focused on breastfeeding, complementary feeding, WASH, healthcare seeking, and preparing meals using products from local agriculture), in order to ensure better health and nutritional status of children [48].

As a longitudinal study conducted over two years, it is possible that living conditions may have improved and, consequently, this could have had some indirect impact on households’ and children’s health and nutrition. For example, all children and household members had important benefits in terms of infections, beyond the measured nutritional outcomes. In addition to receiving the allocated treatment, mothers and caregivers also had the opportunity to be in close contact with the study team (even those living in remote places with poor road access), giving them the opportunity to share their doubts and questions, and to receive the support each time they were followed up in the community. Several authors describe spillover effects in communities [49]. Others, in other age groups, have also pointed out that treating one infected child can also protect nearby children (and adults) who share a latrine or an outdoor bathroom [50]. Moreover, reducing infections in one child benefits the entire community—and neighboring ones as well—by reducing overall infections. From an economic perspective, the cost–benefit and cost-effectiveness aspects of deworming programs are well-established [51]. However, if the “one-size-fits-all solution” brings benefits, despite being more expensive, the TT approach may also provide remarkable benefits across time—especially at the local level, where children can be followed up. This study was implemented via the Dande Health and Demographic Surveillance System [52], and the houses were georeferenced; thus, subsequent studies are feasible to accompany our children and families in the future.

### 4.5. Marginal Models for Binary Longitudinal Data: Strengths and Limitations

Strengths and limitations of the longitudinal study were previously reported [21]. Implementing longitudinal studies is a crucial step to obtain nutritional data on children over a given period in order to make informed decisions. Nevertheless, research on malnutrition in Angola is predominantly cross-sectional. Revisiting the previous study [21] is an attempt to explore longitudinal data, since it is essential to better understand the long-term effects of new treatments and interventions on nutritional outcomes widely used in decision making. Additionally, longitudinal studies are challenging to implement and conduct, and require the long-term availability of material resources and well-trained multidisciplinary professionals qualified to collect nutritional, sociodemographic, and clinical data during different follow-up assessments. This emphasizes the importance of (re)exploring data collected through different statistical approaches, because sometimes relevant findings only appear in one of them. There is an absence of longitudinal data reporting the prevalence of wasting in pre-school children. Demographic surveys are commonly conducted to collect nutritional data on a specific time period using cross-sectional methods, and considering only the moderate-to-severe levels of wasting. However, they do not include the dynamic nature of variation in wasting over time at the individual level.

This was a longitudinal four-arm randomized parallel trial with two years of community follow-up that involved intense logistical organization of a whole community, with the participation of several local health entities, training of logistical and health professionals, and adequate infrastructure, in order to ensure adequate data collection and laboratory analysis. From this point of view, the advantages of performing this secondary analysis of existing nutritional data and an original analysis of primary data on anemia were the low cost, time-saving, and having total and free access to the primary data that allowed the transformation from continuous variables into binary outcomes for nutritional data [53]. However, converting continuous variables into binary variables implies loss of information, which is a limitation from the statistical point of view, but in health studies we need to balance this with the gain of information related to crucial indicators for official statistics and health planning. The sample size was not calculated considering binary data, since the original primary outcome was continuous. In our proposed statistical analysis, we opted for fitting marginal models for binary longitudinal data. These models, also called population-average models, are an alternative approach to the subject-specific models that are often employed in longitudinal analysis. Our choice was due to the primary aims of the clinical trial, which aimed to interpret the results in terms of the involved community rather than a specific child. The process by which repeated measures of binary outcomes are analyzed using population-average and subject-specific logistic regression models is illustrated elsewhere [54], providing some guidance on how to choose between the two approaches by using data from the British Household Panel Survey.

Applying longitudinal models with second-order dependence structures is crucial with respect to the nature of this type of data, where a strong correlation between successive measures should be always considered. Table 3 shows that models without considering serial dependence (independence structure) present higher AIC values compared to models considering a first- or a second-order serial dependence. In the research context, dominated by cross-sectional studies, these findings only emerge from longitudinal studies, which are rare—especially in Angola. Although the clinicians assume the permanently poor status of these children, statistical evidence is needed to support and monitor these children.

## 5. Conclusions

This longitudinal binary analysis with different dependence structure models suggests the potential benefit of testing and treating children with intestinal parasites (A3) for wasting, compared with deworming at the individual level (A1), when considering the mild-to-severe forms. Moreover, it also shows an overall decreased effect on anemia and diarrhea. This study highlights the importance of longitudinal studies using different methods of statistical analysis to assess the progress of stunting and wasting after a given intervention, while also including their mild forms. High percentages of mild malnutrition found in this local setting, omitted in the official indicators, highlight the importance of data trend-tracking, reinforcing the role of constant nutritional surveillance, in order to prevent these children from progressing to more severe forms of malnutrition over time.

## Figures and Tables

**Table 1:** The numbers (*n*) and the percentages (%) of children with mild-to-severe (Mild-to-Sev) and moderate-to-severe (Mod-to-Sev) stunting, by follow-up assessment.

Stunting										
Arms	A1		A2		A3		A4		Total	
Severity	Mild- to-Sev	Mod- to-Sev	Mild- to-Sev	Mod- to-Sev	Mild- to-Sev	Mod- to-Sev	Mild- to-Sev	Mod- to-Sev	Mild- to-Sev	Mod- to-Sev
Follow-Up (Fu)	*n* (%)	*n* (%)	*n* (%)	*n* (%)	*n* (%)	*n* (%)	*n* (%)	*n* (%)	*n* (%)	*n* (%)
Fu1	21 (72.4)	7 (24.1)	21 (67.7)	11(35.5)	21 (67.7)	8 (25.8)	20 (66.7)	11 (36.7)	83 (68.6)	37 (30.6)
Fu2	15 (51.7)	7 (24.1)	20 (64.5)	10(32.3)	21 (67.7)	7 (22.6)	20 (66.7)	10 (33.3)	76 (62.8)	34 (28.1)
Fu3	15 (51.7)	8 (27.6)	17 (54.8)	6 (19.4)	20 (64.5)	9 (29.0)	18 (60.0)	11 (36.7)	70 (57.9)	34 (28.1)
Fu4	15 (51.7)	7 (24.1)	16 (51.6)	7 (22.6)	19 (61.3)	7 (22.6)	16 (53.3)	10 (33.3)	66 (54.5)	31 (25.6)
Fu5	15 (51.7)	7 (24.1)	17 (54.8)	6 (19.4)	17 (54.8)	8 (25.8)	14 (46.7)	9 (30.0)	63 (52.1)	30 (24.8)
Fu6	13 (44.8)	6 (20.7)	15 (48.4)	6 (19.4)	17 (54.8)	7 (22.6)	14 (46.7)	8 (26.7)	59 (48.8)	27 (22.3)

**Table 2:** The numbers (*n*) and the percentages of children (%) with mild-to-severe (Mild-to-Sev) and moderate-to-severe (Mod-to-Sev) wasting over the follow-up period.

Wasting										
Arms	A1		A2		A3		A4		Total	
Severity	Mild- to-Sev	Mod- to-Sev	Mild- to-Sev	Mod- to-Sev	Mild- to-Sev	Mod- to-Sev	Mild- to-Sev	Mod- to-Sev	Mild- to-Sev	Mod- to-Sev
Follow-Up (Fu)	*n* (%)	*n* (%)	*n* (%)	*n* (%)	*n* (%)	*n* (%)	*n* (%)	*n* (%)	*n* (%)	*n* (%)
Fu1	8 (27.6)	1 (3.4)	7(22.6)	2 (6.5)	1 (3.2)	0 (0.0)	4 (13.3)	1 (3.3)	20 (16.5)	4 (3.3)
Fu2	7 (24.1)	1 (3.4)	3 (9.7)	0 (0.0)	3 (9.7)	0 (0.0)	5 (16.7)	0 (0.0)	18 (14.9)	1 (0.8)
Fu3	7 (24.1)	1 (3.4)	4 (12.9)	1 (3.2)	2 (6.5)	1 (3.2)	4 (13.3)	2 (6.7)	17 (14.0)	5 (4.1)
Fu4	5 (17.2)	1 (3.4)	4 (12.9)	1 (3.2)	3 (9.7)	1 (3.2)	6 (20.0)	1 (3.3)	18 (14.9)	4 (3.3)
Fu5	6 (20.7)	2 (6.9)	4 (12.9)	2 (6.5)	2 (6.5)	0 (0.0)	8 (26.7)	0 (0.0)	20 (16.5)	4 (3.3)
Fu6	11 (37.9)	2 (6.9)	7 (22.6)	0 (0.0)	3 (9.7)	0 (0.0)	6 (20.0)	2 (6.7)	27 (22.3)	4 (3.3)

**Table 3:** Model comparison for binary outcomes of stunting and wasting (mild-to-severe and moderate-to-severe degrees).

**Model (M)**	**Structure**	**Effect**	**Mild-to-Severe Stunting**			**Moderate-to-Severe Stunting**		
			**AIC**	**BIC**	**LogLike**	**AIC**	**BIC**	**LogLike**
M1	Independent	Arm + Time	1144.90	1202.32	−567.45	992.79	1050.21	−491.39
M2	Independent	Arm + Time + Age	1096.96	1165.86	−542.48	959.53	1028.43	−473.76
M3	Independent	Arm + Time + Age + Sex	1091.30	1171.69	−538.65	957.82	1038.20	−471.91
M4	MC1-Dependent	Arm + Time	580.72	649.62	−284.36	489.56	558.46	−238.78
M5	MC1-Dependent	Arm + Time + Age	573.31	653.69	−279.65	485.34	565.72	−235.67
M6	MC1-Dependent	Arm + Time + Age + Sex	574.29	666.16	−279.14	485.95	577.81	−234.97
M7	MC2-Dependent	Arm + Time	551.32	**631.70**	−268.66	440.39	**520.78**	−213.20
M8	MC2-Dependent	Arm + Time + Age	**544.83**	636.70	−264.42	**437.23**	529.10	−210.61
M9	MC2-Dependent	Arm + Time + Age + Sex	545.87	649.22	**−263.93**	438.56	541.91	**−210.28**
**Model (M)**	**Structure**	**Effect**	**Mild-to-Severe Wasting**			**Moderate-to-Severe Wasting**		
			**AIC**	**BIC**	**LogLike**	**AIC**	**BIC**	**LogLike**
M1	Independent	Arm + Time	791.52	848.94	−390.76	268.06	325.48	−129.03
M2	Independent	Arm + Time + Age	790.21	859.11	−389.11	267.75	336.65	−127.87
M3	Independent	Arm + Time + Age + Sex	787.04	867.42	−386.52	265.51	345.90	−125.76
M4	MC1-Dependent	Arm + Time	605.60	674.05	−296.80	238.37	307.27	−113.18
M5	MC1-Dependent	Arm + Time + Age	606.96	687.35	−296.48	239.66	320.05	−112.83
M6	MC1-Dependent	Arm + Time + Age + Sex	607.40	699.27	−295.70	239.85	331.71	−111.92
M7	MC2-Dependent	Arm + Time	**580.55**	**660.93**	−283.27	**236.91**	**317.30**	−111.46
M8	MC2-Dependent	Arm + Time + Age	582.25	674.12	−283.12	238.41	330.28	−111.21
M9	MC2-Dependent	Arm + Time + Age + Sex	582.85	686.20	**−282.43**	239.19	342.55	**−110.60**

LogLike: log of the likelihood; MC1: first-order dependence Markov chain structures; MC2: second-order dependence Markov chain structures; AIC: Akaike information criterion; BIC: Bayesian information criterion.

**Table 4:** Selected models for binary outcomes of stunting and wasting (mild-to-severe and moderate-to-severe degrees).

**Model M8**	**Mild-to-Severe Stunting**				**Moderate-to-Severe Stunting**			
**Fixed Effect Parameter**	**Estimate**	**SE**	**OR_adj_**	***p*** **-Value**	**Estimate**	**SE**	**OR_adj_**	***p*** **-Value**
Intercept	3.130	0.9611	-	0.001	1.398	1.0922	-	0.201
A2	0.401	0.4669	1.494	0.390	0.319	0.5323	1.375	0.550
A3	0.348	0.4611	1.417	0.450	−0.009	0.5345	0.991	0.987
A4	0.379	0.4684	1.461	0.418	0.539	0.5308	1.714	0.310
Time	−0.031	0.0082	0.969	<0.001	−0.019	0.0076	0.982	0.015
Age	−0.100	0.0347	0.905	0.004	−0.090	0.0408	0.914	0.027
**Dependence Parameter:**								
log.psi1	4.786	0.3551	-	<0.001	4.971	0.4332	-	<0.001
log.psi2	2.244	0.3856	-	<0.001	3.060	0.4204	-	<0.001
**Model M7**	**Mild-to-Severe Wasting**				**Moderate-to-Severe Wasting**			
**Fixed effect parameter**	**Estimate**	**SE**	**OR_adj_**	***p*** **-value**	**Estimate**	**SE**	**OR_adj_**	***p*** **-value**
Intercept	−0.604	0.3297	-	0.067	−2.331	0.5482	-	<0.001
A2	−0.674	0.4441	0.510	0.130	−0.562	0.7011	0.570	0.423
A3	−1.319	0.4858	0.268	0.007	−1.476	0.8436	0.229	0.080
A4	−0.562	0.4398	0.570	0.202	−0.305	0.6796	0.737	0.654
Time	−0.015	0.0126	0.986	0.248	−0.036	0.0272	0.965	0.190
**Dependence Parameter:**								
log.psi1	3.101	0.3223	-	<0.001	3.106	0.6310	-	<0.001
log.psi2	1.767	0.3252	-	<0.001	1.539	0.7608	-	0.043

SE: standard error, OR_adj_: adjusted odds ratio, A1: Arm 1, A2: Arm 2, A3: Arm 3, A4: Arm 4.

**Table 5:** Anemia and malaria in children, by follow-up.

Clinical Data	Anemia					Malaria—*Plasmodium falciparum*				
Arms	A1	A2	A3	A4	Total	A1	A2	A3	A4	Total
Follow-Up (Fu)	*n* (%)	*n* (%)	*n* (%)	*n* (%)	***n* (%)**	*n* (%)	*n* (%)	*n* (%)	*n* (%)	***n* (%)**
Baseline	16 (59.3)	16 (51.6)	13 (41.9)	19 (63.3)	**64 (53.8)**	4 (13.8)	3 (9.7)	2 (6.5)	2 (6.7)	**11 (9.1)**
Fu1	11 (39.3)	11 (40.7)	8 (29.6)	10 (34.5)	**40 (36.0)**	2 (7.1)	0 (0.0)	2 (7.4)	2 (6.9)	**6 (5.4)**
Fu2	9 (32.1)	7 (25.0)	6 (23.1)	4 (13.8)	**26 (23.4)**	2 (7.1)	0 (0.0)	2 (7.7)	1 (3.6)	**5 (4.5)**
Fu3	9 (32.1)	5 (19.2)	8 (30.8)	10 (34.5)	**32 (29.4)**	2 (7.1)	0 (0.0)	0 (0.0)	1 (3.7)	**3 (2.8)**
Fu4	5 (20.8)	6 (23.1)	6 (24.0)	6 (20.7)	**23 (22.1)**	0 (0.0)	0 (0.0)	2 (8.0)	4 (13.8)	**6 (5.7)**
Fu5	4 (15.4)	7 (26.9)	7 (26.9)	9 (34.6)	**27 (26.0)**	2 (7.7)	1 (3.7)	2 (7.7)	3 (11.5)	**8 (7.6)**
Fu6	9 (33.3)	8 (29.6)	7 (26.9)	7 (25.9)	**31 (29.0)**	3 (11.1)	3 (11.1)	2 (7.7)	1 (3.7)	**9 (8.4)**

**Table 6:** Diarrhea and vomiting in children, by follow-up.

Clinical Data	Diarrhea					Vomiting				
Arms	A1	A2	A3	A4	Total	A1	A2	A3	A4	Total
Follow-Up (Fu)	*n* (%)	*n* (%)	*n* (%)	*n* (%)	***n* (%)**	*n* (%)	*n* (%)	*n* (%)	*n* (%)	***n* (%)**
Baseline	15 (51.7)	10 (32.3)	16 (51.6)	17 (56.7)	**58 (47.9)**	4 (13.8)	7 (22.6)	5 (16.1)	2 (6.7)	**18 (14.9)**
Fu1	5 (17.9)	6 (22.2)	6 (22.2)	6 (20.7)	**23 (20.7)**	2 (7.1)	0 (0.0)	0 (0.0)	0 (0.0)	**2 (1.8)**
Fu2	6 (21.4)	5 (17.2)	5 (20.0)	7 (24.1)	**23 (20.7)**	1 (3.6)	0 (0.0)	0 (0.0)	0 (0.0)	**1 (0.9)**
Fu3	5 (17.9)	6 (21.4)	3 (11.1)	4 (13.8)	**18 (16.1)**	0 (0.0)	0 (0.0)	0 (0.0)	0 (0.0)	**0 (0.0)**
Fu4	5 (20.8)	6 (22.2)	4 (16.0)	3 (10.3)	**18 (17.1)**	1 (4.2)	0 (0.0)	1 (4.0)	0 (0.0)	**2 (1.9)**
Fu5	4 (15.4)	3 (11.1)	1 (3.8)	3 (11.5)	**11 (10.5)**	0 (0.0)	0 (0.0)	0 (0.0)	0 (0.0)	**0 (0.0)**
Fu6	1 (3.7)	2 (7.4)	3 (11.5)	4 (14.8)	**10 (9.3)**	0 (0.0)	0 (0.0)	1 (3.8)	0 (0.0)	**1 (0.9)**

## Data Availability

The data presented in this study are available upon request from the corresponding author. The data are not publicly available due to ethical requirements.

## Abbreviations

(A1): Arm 1; (A2): Arm 2; (A3): Arm 3; (A4): Arm 4; (ALB): albendazole; (Annual-ALB): annual single-dose albendazole; (Annual-ALB*individual level): annual single-dose albendazole provided at the individual level in Arm 1; (Annual-ALB*household level): annual single-dose albendazole provided at the household level in Arm 2; (AIC) Akaike information criterion; (BIC): Bayesian information criterion; (CI): confidence interval; (CISA): Health Research Centre of Angola; (CONSORT): Consolidated Standards of Reporting Trials; (Fu): follow-up; (Fu1): follow-up performed 4 months after participant inclusion; (Fu2): follow-up performed 8 months after participant inclusion; (Fu3): follow-up performed 12 months after participant inclusion; (Fu4): follow-up performed 16 months after participant inclusion; (Fu5): follow-up performed 20 months after participant inclusion; (Fu6): follow-up performed 24 months after participant inclusion; (g/dL): grams per deciliter; (H): helminths; (HAZ): height-for-age Z-score; (HDSS): Dande Health and Demographic Surveillance System; (Mild-to-Sev): mild-to-severe; (Mod-to-Sev): moderate-to-severe; (MC1): first-order Markov chain; (MC2): second-order Markov chain; (M): model; (OR_adj_): adjusted odds ratio; (P): intestinal protozoan parasites; (P + H): intestinal protozoans and helminths; (SDG): Sustainable Development Goals; (STH): soil-transmitted helminth; (t): time; (WASH): water, sanitation, and hygiene; (WHO): World Health Organization; (WHZ): weight-for-height Z-score; (TT): test-and-treat (4TT): four-monthly test-and-treat; (4TT*individual): a four-monthly test-and-treat approach to intestinal parasites provided at the individual level in Arm 3; (4TT*household level): a four-monthly test-and-treat approach to intestinal parasites provided at the household level in Arm 4.

## Appendix A

**R code for model M8 considering mild-to-severe stunting:**

$$logit\;\left[E\left(Y_{it}\right)\right]=\beta_{1}+\beta_{2}\;arm2_{it}+\beta_{3}\;arm3_{it}+\beta_{4}\;arm4_{it}+\beta_{5}\;time_{it}+\;\beta_{6}\;age_{i}$$

s_MC2_m2 < −bild(stunting ~ arm + time + age, data = dataset, id = “subject”,

+ start = NULL, dependence = “MC2”)

summary(s_MC2_m2)

Call: bild(formula = stunting ~ arm + time + age, data = dataset, id = “subject”,

start = NULL, dependence = “MC2”)

Number of profiles in the dataset: 121

Number of profiles used in the fit: 121

Dependence structure: MC2

Log likelihood: −264.4161

AIC: 544.8323

Fixed effects:

        Estimate    Std. Error    z value   *p*-value

(Intercept)        3.12994834   0.961143713   3.256    0.001128

arm2        0.40115452   0.466917154   0.859    0.390255

arm3        0.34825897   0.461104865   0.755    0.450087

arm4        0.37941459   0.468406038   0.810    0.417933

time         −0.03148195        0.008164289   −3.856         0.000115

age           −0.09953121        0.034658792   −2.872         0.004082

Dependence parameter:

        Estimate    Std. Error    z value      *p*-value

log.psi1      4.785789    0.3551437    13.476        0.000

log.psi2      2.244194    0.3856198    5.820           0.000

## Appendix B

**Residual analysis for model M8 considering mild-to-severe stunting.**
